# Leptin and Cancer: Updated Functional Roles in Carcinogenesis, Therapeutic Niches, and Developments

**DOI:** 10.3390/ijms22062870

**Published:** 2021-03-11

**Authors:** Tsung-Chieh Lin, Michael Hsiao

**Affiliations:** 1 Genomic Medicine Core Laboratory, Department of Medical Research and Development, Chang Gung Memorial Hospital, Linkou 333, Taiwan; tclin1980@cgmh.org.tw; 2 Genomics Research Center, Academia Sinica, Taipei 115, Taiwan; 3 Department of Biochemistry, College of Medicine, Kaohsiung Medical University, Kaohsiung 807, Taiwan

**Keywords:** leptin, prognosis, cancer progression

## Abstract

Leptin is an obesity-associated adipokine that is known to regulate energy metabolism and reproduction and to control appetite via the leptin receptor. Recent work has identified specific cell types other than adipocytes that harbor leptin and leptin receptor expression, particularly in cancers and tumor microenvironments, and characterized the role of this signaling axis in cancer progression. Furthermore, the prognostic significance of leptin in various types of cancer and the ability to noninvasively detect leptin levels in serum samples have attracted attention for potential clinical applications. Emerging findings have demonstrated the direct and indirect biological effects of leptin in regulating cancer proliferation, metastasis, angiogenesis and chemoresistance, warranting the exploration of the underlying molecular mechanisms to develop a novel therapeutic strategy. In this review article, we summarize and integrate transcriptome and clinical data from cancer patients together with the recent findings related to the leptin signaling axis in the aforementioned malignant phenotypes. In addition, a comprehensive analysis of leptin and leptin receptor distribution in a pancancer panel and in individual cell types of specific organs at the single-cell level is presented, identifying those sites that are prone to leptin-mediated tumorigenesis. Our results shed light on the role of leptin in cancer and provide guidance and potential directions for further research for scientists in this field.

## 1. Introduction

**Figure 1:**
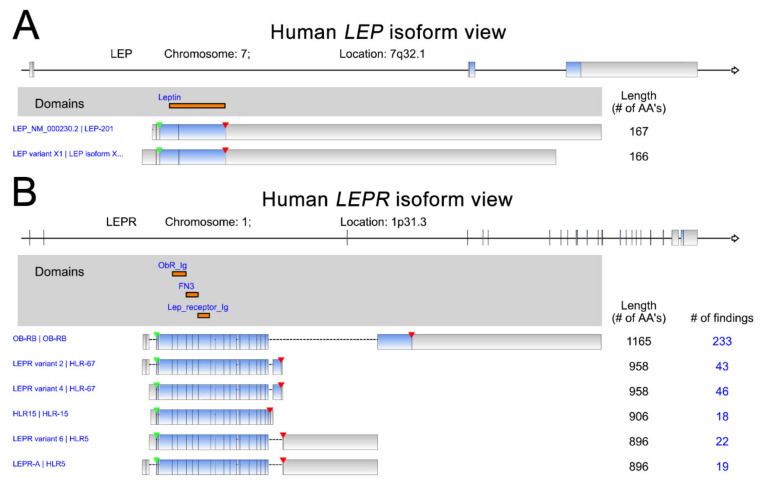
Human LEP (**A**) and LEPR (**B**) isoforms. The data were retrieved and analyzed from RefSeq. The protein domains of various isoforms are indicated in orange. The start of transcription and stop codon position are indicated by green and red arrowheads, respectively.

Leptin (*LEP*), a protein hormone secreted by adipose tissues, primarily functions as the ligand of leptin receptor (*LEPR*) to regulate appetite and energy expenditure [1,2]. Leptin plays critical roles in the modulation of processes involving in the hormones synthesis, blood pressure, reproduction, osteogenesis, hematopoiesis, angiogenesis, and immunity [3]. Moreover, leptin is also uncovered to participate in the pathophysiology of energy metabolism [4], endocrine diseases [5], neurovascular diseases [6], or obesity and metabolism-associated diseases [7]. Leptin is encoded by the OB gene on chromosome 7. The 2 LEP isoforms and 6 LEPR isoforms are shown with the protein domains indicated (Figure 1). The leptin receptor, encoded by *LEPR*, is a member of the class 1 cytokine receptor family and has been indicated to play critical roles in the pathogenesis of many malignant cancer types [1,8,9]. The downstream effects of leptin signaling can induce malignancies via the activation of specific signaling pathways in cancer cells [10,11,12]. Recent studies indicate that leptin receptors are highly abundant in many cancer types [13,14,15]. Accumulated experimental results have highlighted the role of leptin–leptin receptor signaling in promoting several processes linked to cancer progression, including cell proliferation, metastasis, angiogenesis and chemoresistance [16,17,18,19]. In this review article, we integrate and summarize the current literature on this topic, focusing on evidence demonstrating leptin/leptin receptor expression levels in a broad range of cancer types together with its biological effects on the regulation of several critical processes related to cancer progression. In addition to the biological function of the leptin axis in cancer, its clinical and prognostic significance in multiple cancer types is illustrated.

## 2. Leptin and Leptin Receptor Expression in Cancer

**Figure 2:**
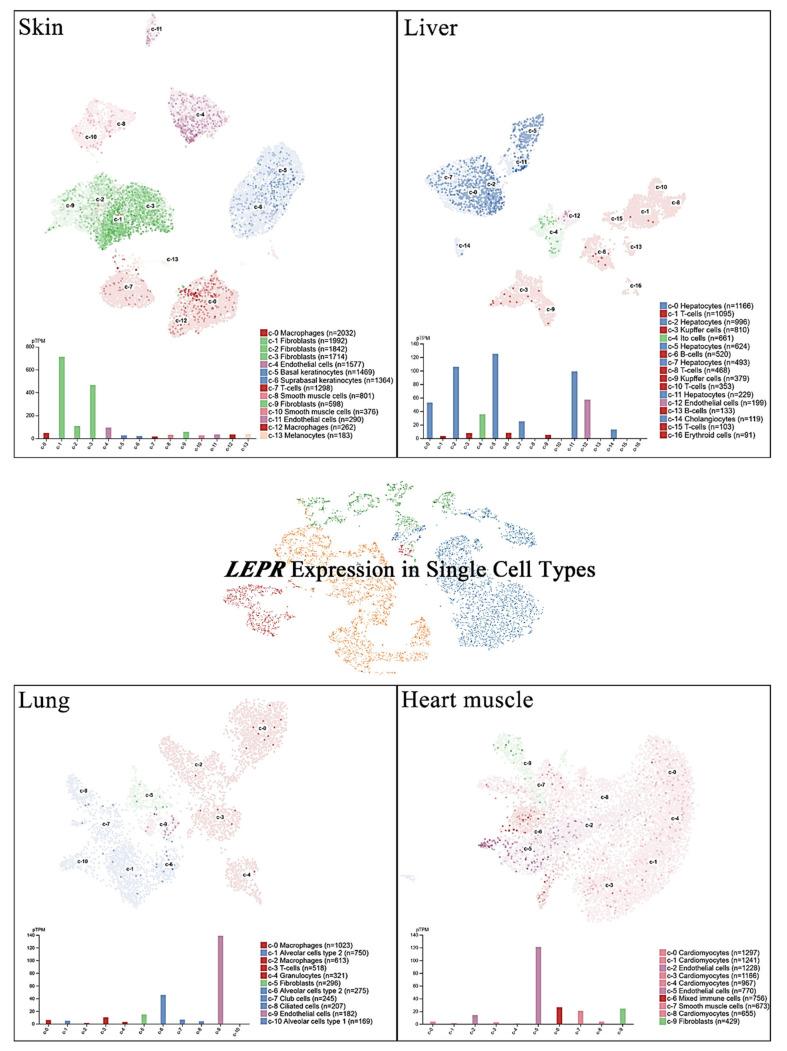
LEPR expression in single cells of different types. The LEPR expression level was analyzed by single-cell RNA sequencing (scRNA-seq) in various human tissues. The RNA expression levels in the cell type clusters identified in each tissue were visualized by a UMAP plot of single cells (**top**) and in a bar chart (**bottom**). The read counts were normalized to transcripts per million protein-coding genes (pTPM) for each cluster.

Single-cell RNA sequencing (scRNA-seq) has become a powerful tool to delineate the composition of different cell types or states in a given tissue on the basis of sets of differentially expressed genes [20,21,22,23]. Recently, scRNA-seq of normal tissue led to the discovery of specific biomarkers in multiple cell types that might contribute to cancer development [24]. A new cell-type atlas with publicly available genome-wide expression scRNA-seq data of 192 individual cell-type clusters from 13 different human tissues was launched in November 2020 (The Human Protein Atlas, https://www.proteinatlas.org/, accessed on January 2021) [25]. The leptin receptor expression in the four normal tissues with the highest leptin receptor levels, namely, the skin, lung, liver, and heart, is shown at the single-cell scale (Figure 2). Relatively high leptin receptor expression was detected in fibroblasts, endothelial cells, and macrophages in skin tissue. Hepatocytes, Ito cells, endothelial cells and cholangiocytes in the liver all showed leptin receptor expression. In addition, leptin receptor expression was specifically detected in alveolar type 2 cells and endothelial cells compared with other lung cell types. In the heart, leptin receptor expression was found in endothelial cells, mixed immune cells, smooth muscle cells, and fibroblasts, but not in cardiomyocytes. These observations further suggest potential sites of leptin-mediated signaling that may play roles in tumorigenesis.

Cancer cells and the tumor microenvironment expressing leptin and leptin receptors suggest that the potential leptin autocrine/paracrine signaling loop could affect tumor progression. A total of 32 blood samples from colorectal cancer patients and 25 healthy subjects were analyzed for serum levels of leptin. Leptin was significantly higher in patients than in controls (*p* < 0.05) [26]. An immunohistochemical analysis of bone metastatic tissue of breast cancer further showed that the leptin receptor was prevalently expressed in the cytosol and the nuclei of metastatic cells, whereas leptin was detected in both metastatic cells and stromal cells [27]. A cohort of gastric cancer enrolling 117 newly diagnosed and untreated patients was studied. The results indicated that LEPR methylation levels were significantly lower in tumor samples than in adjacent (5 cm away) nontumor samples, suggesting the potentially high expression level of leptin receptor in gastric cancer [28]. An immunohistochemistry study using a tissue microarray of bladder cancer showed that strong leptin expression tended to be present more often in tumors than in benign tissues [29]. Higher RNA levels of both leptin and leptin receptors were found in prostate cancer patients than in healthy controls in a study including 176 men [30]. Leptin levels in patients with endometrial cancer were significantly higher than those in the control group [31].

**Figure 3:**
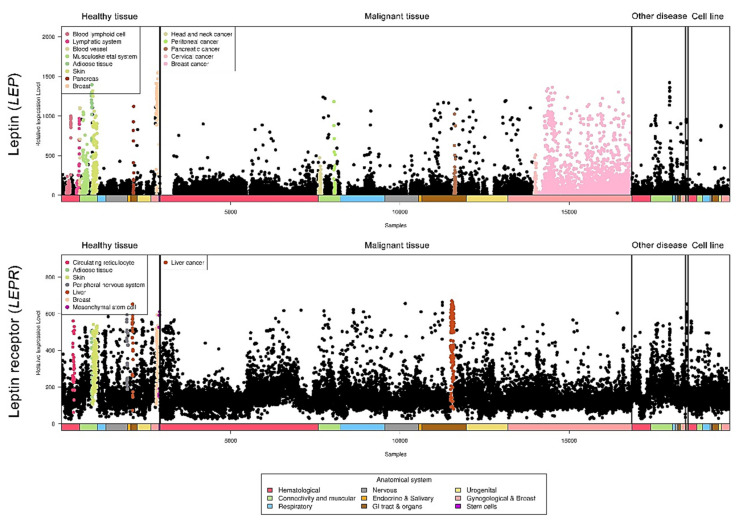
LEP and LEPR expression in a pancancer panel. In a pancancer dataset, LEP and LEPR expression levels were presented separately in various cancer types. The colored dots represent individual patients with higher expression of the indicated molecule among all cancer types. The raw data were retrieved from the online IST database.

Relative leptin and leptin receptor expression data from different types of cancers were retrieved from a public database (https://ist.medisapiens.com/, accessed on January 2021) (Figure 3). The relative leptin expression level is high in head and neck cancer, peritoneal cancer, pancreatic cancer, cervical cancer, and breast cancer. In addition, leptin receptor expression is relatively high in liver cancer, indicating the potential pathological roles of this axis in cancers.

## 3. LEP Somatic Mutations and Cancer

Genetic variants of the *LEP* gene in cancer patients have been reported in several studies. In a case-control study with 380 gastric cancer patients and 465 normal controls, the *LEP* G19A polymorphism was statistically correlated with a decreased risk of cancer susceptibility [32]. Similar results were published exploring this association in a bladder cancer cohort enrolling 355 cancer cases and 435 controls (all Chinese Han). The *LEP* G19A polymorphism was reported to be significantly associated with lower cancer risk, smaller tumor size, less node metastasis, and less distant metastasis [33]. In a colorectal cancer cohort of 1003 cases and 1303 matched controls, *LEPR* rs6588147, rs1137101, and *LEP* rs2167270 polymorphisms were found to be associated with a decrease in cancer risk, whereas *LEPR* rs1137100 was associated with cancer susceptibility [34]. In addition, a comprehensive encyclopedia of somatic mutation calls in cancer patients has been published by TCGA [35]. *LEP* mutation signatures in several cancer types are shown (Table 1).

### 3.1. Leptin and Ovarian Cancer

The influence of leptin on chemoresistance in epithelial ovarian cancer has been studied. Treatment with exogenous leptin decreased paclitaxel/docetaxel-induced G2/M phase cell cycle arrest in ovarian cancer cells [36]. However, experimental results from another research group showed that serum levels of leptin were not correlated with the response to paclitaxel/carboplatin therapy in ovarian cancer patients [37]. Leptin-induced ovarian cancer cell invasion via overexpression of MMP7, MMP9, and uPA was reported, and the involvement of the estrogen-independent role of ERα in regulating the phenotype was indicated [38]. In addition, leptin was found to elicit matrix metalloproteinase 7 expression, leading to increased ovarian cancer cell invasion via ERK and JNK pathway activation [39]. Furthermore, the RhoA-ROCK signaling axis was verified to mediate leptin-promoted uPA expression to promote cell invasion in ovarian cancer cells [40]. Leptin has also been shown to regulate cancer cell proliferation. The biological effect of leptin on inducing ovarian cancer cell growth was mediated by an increase in cyclin D1 and Mcl-1 expression after the activation of the PI3K/Akt and MEK/ERK1/2 signaling axes [41]. Treatment with recombinant leptin was found to contribute to ovarian cancer cell migration, invasion, peritoneal metastasis and epithelial-mesenchymal transition (EMT), and the malignant phenotypes were caused by activation of the PI3K/Akt/mTOR signaling pathway [42]. The overexpression of histone deacetylases (HDACs) was known to elicit carcinogenesis, and leptin receptor antagonists, SHLA and Lan2, were shown to eliminate the effect induced by leptin in ovarian cancer [43].

### 3.2. Leptin and Brain Tumor

Leptin and its receptor were reported to be highly expressed in brain tumors, and the patients with the tissues displaying those biomarkers associated with the degree of malignancy [44]. The roles of the leptin-leptin receptor signaling axis in brain tumor progression were evaluated. The biological interaction of leptin with Notch signaling pathways was found to be required for glioblastoma multiforme (GBM) development and progression. The activation of leptin downstream effectors induced growth and motility in GBM cells, and the induced effects on GBM cancer cells were inhibited by the selective leptin antagonist as well as by the specific inhibitor of Notch signaling, suggesting that leptin/Notch crosstalk may be a potential novel target for GBM therapeutics [45]. Leptin treatment alone and cotreatment with secreted phospholipase A2-IIA (sPLA2-IIA) led to the phosphorylation activation of the Src/ERK/Akt/mTOR/p70S6K/rS6 pathway, thereby inducing cell proliferation in the human astrocytoma cell line 1321N1 [46]. In addition, leptin signaling is linked to angiogenesis in glioblastoma, and the correlation of leptin receptors with vasculogenic mimicry (VM) has been identified [47]. The observation of the leptin signaling axis in inducing angiogenesis was then reported by another group, which further showed the reverse effect of Aca1 (leptin receptor antagonist) on endothelial cell tube formation activity [48]. Moreover, ectopic leptin receptor overexpression resulted in temozolomide (TMZ) resistance in glioblastoma, and the involvement of stem/progenitor cell properties and STAT3 signaling was indicated [49].

### 3.3. Leptin and Breast Cancer

The reciprocal interaction of the adipose microenvironment and breast cancer tissue is a critical factor in promoting cancer cell migration [50]. Experimental results showed that adipose cells led to a decrease in the viability of myoepithelial cells (MECs), which are known as tumor suppressor cells that block the transition from ductal carcinoma in situ to invasive carcinoma. Both leptin amplification and the disruption of genes involved in extracellular matrix maintenance were observed during this stage [51]. The effect of the adipokine leptin on normal mammary epithelial cells was investigated. Leptin treatment triggers EMT-like features, including migration speed, via the interdependent activity of leptin receptor and Ca^2+^ channel-mediated myosin light chain kinase-2 (MLC-2) phosphorylation [52]. An orthotopic implantation metastasis model further revealed that plasminogen activator inhibitor-1 (PAI-1) is required for breast cancer metastasis. Mechanistically, the upregulation of PAI-1 expression was elicited by leptin-LEPR-miR-34a axis-mediated STAT3 signaling activation [53]. Leptin also elicits metabolic reprogramming in breast cancer. This function was found to be accompanied by autophagy activation and SREBP-1 induction, leading to stimulated cell proliferation [54]. A breast cancer cohort including 106 cases was studied. Higher leptin receptor expression levels were associated with an increased incidence of bone metastasis in breast cancer patients. Leptin addition also activated the SDF-1/CXCR4 axis to promote invasive behavior in the breast cancer cell lines MCF-7 and SK-BR-3 [55]. The loss of LEPR expression in breast cancer was also observed to modulate the tumor microenvironment. With LEPR loss, cancer cells exhibited a less aggressive phenotype, and macrophage recruitment was abolished; the phagocytic activity and cytokine production of macrophages also appeared to decrease [56]. Cotreatment with leptin and adipose tissue-derived fibroblast growth factor-2 (FGF2) was reported to induce the malignant transformation of breast epithelial MCF-10A cells, and this effect was found to be attenuated by ruxolitinib and AG490, two separate inhibitors of Jak2, which is downstream of the leptin receptor, suggesting the critical interplay of leptin, leptin receptor and Jak2 in cancer progression [57]. The inhibitory effects of globular adiponectin on leptin-promoted inflammasome activation and tumor growth were further verified in breast MCF-7 cell and xenograft models via mechanisms including HO-1 induction and ER-α signaling modulation [58]. In addition, tamoxifen was found to increase the expression of leptin receptor in breast cancer cell lines, leading to the decrease of drug sensitivity in inhibiting cell proliferation [59].

### 3.4. Leptin and Liver Cancer

The role of the leptin-leptin receptor axis in triggering hepatic tumor malignancy was identified. Leptin was discovered to elicit the phosphorylation activation of STAT3, ERK1/2, and Akt in liver cancer cells, thereby enhancing cell proliferation and migration ability. In particular, the adaptor protein APPL1 directly interacts with STAT3 and leptin receptors to enhance the aforementioned phenotypes in the human hepatocellular carcinoma cell line HepG2 [60]. In addition, leptin receptor was characterized to be expressed in various types of cancer, suggesting biological functions for leptin outside of appetite regulation. Overexpression of leptin receptor was significantly associated to the unfavored TNM status in hepatocellular carcinoma [61]. In another report of human hepatocellular carcinoma, leptin/leptin receptor expression was observed in both tumor and endothelial cells, in parallel to the degree of angiogenesis [62]. The underlying mechanisms of LEPR in liver cancer metastasis were addressed. LEPR could enhance proliferation, migration, and invasion and inhibit apoptosis in lymphatic metastasis of hepatocellular carcinoma by directly interacting with ANXA7 [63]. Interestingly, the leptin-derived peptide (mimetic), OB3, was able to abolish leptin-induced cell proliferation by reducing phosphoinositide 3-kinase (PI3K) activation and the expression of proinflammatory genes in hepatocellular carcinoma cells [64]. The opposite function of leptin in cell proliferation was reported in rat hepatocellular carcinoma, which occurs via a p38-MAPK-dependent signaling pathway to attenuate serum-induced H4IIE cell proliferation [65].

### 3.5. Leptin and Colorectal Cancer

Notably, a study in colon cancer demonstrated that leptin could upregulate miR-4443 to restrain TRAF4 and NCOA1 expression, leading to decreased cancer invasion [66]. In addition, leptin stimulation was found to promote the migration and invasion of cultured HCT-116 cells, tumor growth in the xenograft model and the upregulation of SIRT1. These effects were abolished by the addition of the SIRT1 inhibitor sirtinol, indicating the critical involvement of SIRT1 in obesity-associated colon carcinogenesis [67]. The critical role of leptin receptor expression in the proliferation of colorectal carcinoma has also been evaluated in the clinic. The absence of leptin receptor expression was found to be associated with a low tumor proliferation index in 94.1% of cases [68]. In a cohort study of 75 colorectal carcinoma patients, elevated LEPR expression was accompanied by the observation of neoangiogenesis and an increase in metastatic potential [69]. The carcinogen-induced aberrant crypt foci (ACF) was reduced in the intestinal epithelium-specific leptin receptor conditional knockout mice accompanied by the activation of STAT3 signaling, indicating its impact on tumorigenesis [70]. Interestingly, the unique ability of leptin to target the leptin receptor was exploited to enhance drug delivery in colon cancer. PEGylated liposomal doxorubicin decorated with a leptin-derived peptide (Lp31) showed improved uptake by and cytotoxicity against C26 cells. The results of animal experiments revealed the suppression of tumor growth, consistent with the increased doxorubicin concentration in tumor tissue [71]. A similar outcome was published by another group using leptin-derived peptide (LP16, 91–110 of leptin) to reduce tumor growth in a C26 colon carcinoma tumor-bearing mouse model [72].

### 3.6. Leptin and Lung Cancer

The expressions of leptin and leptin receptor were significantly higher in non-small-cell lung cancer (NSCLC) tissues than in normal lung tissues [73]. In addition, a cohort of 71 patients with early-stage NSCLC was studied. The mean serum leptin level was found to be significantly higher in patients with adenocarcinoma than in those with the squamous cell subtype, suggesting that measuring serum leptin could be a noninvasive method for pathological diagnosis [74]. In clinical data, the correlation of leptin and leptin receptor expression with bone metastasis was detected in pulmonary adenocarcinoma patients [75]. Leptin was able to induce EMT in the lung cancer cell line A549, thereby promoting cell migration, invasion, and tumorigenesis. The study also provided evidence that leptin-induced malignancies were driven through the activation of the ERK signaling axis [76]. In a study of brain metastasis of lung adenocarcinoma, activation of leptin signaling was highlighted in the context of the lnc-REG3G-3-1^high^/miR-215-3p^low^ axis [77]. The assessment of leptin drug resistance showed that bone marrow-derived mesenchymal stem cells could release leptin to induce erlotinib resistance in lung adenocarcinoma cells by activating IGF-1R signaling in a hypoxic environment, suggesting a predictive role of leptin expression for therapeutic response [78]. Similar observations showed that leptin overexpression decreased the cisplatin-mediated ER stress unfolded protein response pathways PERK and ATF6 to promote lung adenocarcinoma A549 cell proliferation [79]. In addition, a carcinogenic role of leptin in the tumor microenvironment was discovered. Leptin secretion from cancer-associated fibroblasts (CAFs) could trigger the proliferation and migration of NSCLC cells through the activation of the PI3K-AKT and MAPK-ERK signaling axes in a paracrine manner [80].

### 3.7. Leptin and Pancreatic Cancer

Oncogenic hypoxia inducible factor (HIF)-1α was identified to bind directly to hypoxia-responsive elements (HREs) located in the LEPR gene promoter (-828/-832), thereby activating downstream transcriptional events in pancreatic cancer cells, suggesting the potential significance of the leptin receptor-mediated axis during hypoxia [81]. In a study of pancreatic cancer, the results demonstrated that exogeneous leptin could enhance cell proliferation, glucose uptake and lactate production in a dose-dependent manner, and that this was accompanied by elevated expression of the glycolytic enzymes hexokinase II and glucose transporter 1, suggesting the potential involvement of glucose metabolism in pancreatic cancer progression [82]. In addition, simultaneously high leptin receptor and MMP13 production exhibited a positive correlation with TNM status in pancreatic cancer patients. Leptin-induced cancer cell migration, invasion and metastasis were also observed in a pancreatic orthotopic model [83]. Another research group further indicated that leptin could promote cancer progression and increase ABCB1 protein synthesis in pancreatic cancer [84]. Emerging studies have referred to the participation of the leptin signaling axis in drug resistance. Leptin levels in serum were found to be higher in patients with pancreatic adenocarcinoma and correlated with resistance to gemcitabine chemotherapy [85]. Furthermore, leptin was reported to elicit chemoresistance in pancreatic ductal adenocarcinoma, and the results indicated that gemcitabine resistance develops via miR-342-3p upregulation-dependent inhibition of KLF6 signaling in cancer cells [86]. Leptin also decreased 5-fluorouracil (5-FU) cytotoxicity and promoted cell proliferation, colony formation ability, and stem cell pluripotency. The expression of EMT markers, drug efflux proteins (ABCC5, ABCC11) and Notch appeared to be upregulated [87]. The role of leptin receptor in pancreatic cancer was also reported. Reduction of leptin receptor by shRNA knockdown was observed to partially abrogate tumor growth in obese mice of orthotopic murine pancreatic cancer model [88].

### 3.8. Leptin and Other Types of Cancer

**Figure 4:**
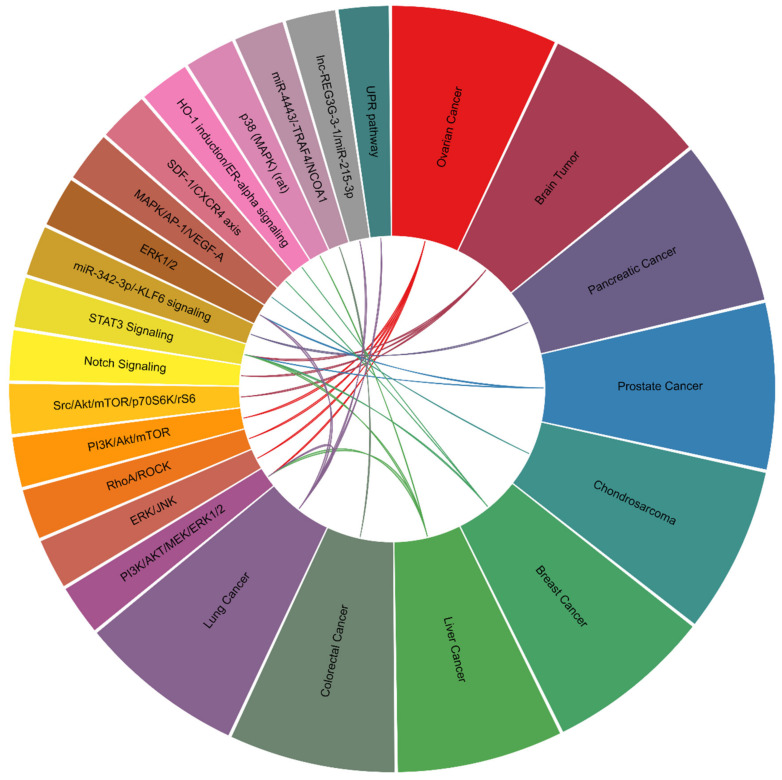
Signaling axes modulated by leptin/leptin receptor.

In prostate cancer, an increase in DU145 cell proliferation and invasion and a decrease in cell apoptosis due to ERK1/2 signaling activation after leptin treatment were reported [89]. The effect of leptin on prostate cancer progression was assessed in DU-145 and PC3 cell lines. Leptin treatment appeared to promote cancer cell migration and EMT transition by activating the STAT3 pathway [90]. Findings in human gallbladder cancer suggested the requirement of leptin-leptin receptor signaling axis activation in cancer progression because leptin increased cell proliferation via the leptin receptor [91]. In myeloma, bortezomib treatment-induced cytotoxicity was attenuated by leptin, accompanied by the upregulation of cyclin D1 and Bcl-2 and downregulation of caspase 3 [92]. In chondrosarcoma, there is significant evidence that leptin can induce VEGF-C expression and its secretion, contributing to the lymphangiogenesis of human lymphatic endothelial cells by repressing miR-27b [93]. Leptin-dependent regulation of tube formation in endothelial progenitor cells was shown in a chondrosarcoma cell study. MAPK signaling was activated to induce AP-1 binding to the VEGF-A promoter and initiate transactivation upon stimulation of the leptin-leptin receptor signaling axis in cancer cells [94]. In addition, a correlation analysis further indicated the positive association of leptin and leptin receptor levels with lymph node metastasis in endometrial cancer patients [95]. A study aiming to counteract leptin-mediated cancer metastasis was reported. Adiponectin was found to attenuate leptin-elicited SPEC-2 endometrial cancer metastasis by inhibiting the JAK/STAT3 pathway via AMPK activation [96]. In a study of squamous cell carcinoma of the skin, leptin receptor expression as evaluated by immunostaining was significantly correlated with poor differentiation, proliferation index and tumor histologic grade [97]. Another clinical study revealed that cutaneous melanoma patients with higher leptin levels in serum samples also had a high risk of sentinel lymph node metastasis [98]. In addition, elevation of VEGF-A expression by leptin was reported in melanoma [99]. The demonstrations of main biological effects (Table 2) and signaling axes (Figure 4) induced by leptin/leptin receptor in different cancer types were shown.

## 4. Correlation of Leptin and Leptin Receptor Levels with Clinical Outcomes in Cancer

The clinical association of leptin and leptin receptor expression with cancer patient outcomes has been explored, and the positive association of leptin with hepatocellular carcinoma risk was recently reported [100]. Interestingly, the specific cellular localization of leptin shows prognostic power; its nuclear expression was identified to be significantly associated with overall survival in patients with clear cell renal cell carcinomas [101]. In bladder cancer, a multivariate analysis revealed a higher risk of progression (HR = 5.148, 95% CI = 1.190–22.273; *p* = 0.028) in patients with leptin-positive muscle-invasive tumors [29]. In addition, a positive association between LEPR mRNA expression and unfavorable prognosis was found in prostate cancer [90]. A similar positive correlation was also discovered between leptin/leptin receptor overexpression and distant metastasis in a cohort of 176 prostate cancer patients [30]. The expression levels of leptin and its receptor were both found to be associated with unfavorable prognosis in patients with endometrial cancer (3-year survival rate) [95]. A cohort study enrolling ovarian cancer patients indicated a significant correlation of leptin and leptin receptor coexpression with shorter survival time [102]. In addition, serum leptin and leptin receptor RNA expression showed a positive association with cancer recurrence and mortality in triple-negative breast cancer [103]. Leptin levels also exhibited a positive correlation with poor prognosis in ovarian cancer [42], and high leptin levels were found in multiple myeloma patients and to correlate with clinical stage [92]. The leptin receptor also displays prognostic significance in the clinic. Patients with glioblastoma showing poor prognosis have high leptin receptor levels [47]. In upper tract urothelial carcinomas, leptin receptor expression was associated with unfavorable prognosis for recurrence-free survival (*p* = 0.09) and cancer-specific survival (*p* = 0.01) by log-rank test. Cox regression analysis further characterized leptin receptor expression as an independent biomarker predicting poor survival [104]. In contrast, results illustrating the negative correlation of leptin or leptin receptors with cancer progression have also been reported. Immunohistochemical staining showed that cytoplasmic leptin was less frequently observed in breast cancers with unfavorable prognosis [105]. In nonmetastatic renal cell carcinoma, a relatively high disease recurrence rate and low recurrence-free survival rate were associated with high CpG methylation in the *LEPR* gene promoter region [106]. *LEP* RNA expression profiles investigated by RNA-seq and microarray approaches have been released together with clinical patient follow-up data from public databases, including The Human Protein Atlas/The Pathology Atlas [25,107,108,109,110] and the Kaplan–Meier plotter database [111], which illustrate the prognostic value of *LEP* in specific cancer types (Table 3). *LEP* was an unfavorable prognostic marker in cohorts of glioma, lung cancer, colorectal cancer, renal cancer, ovarian cancer, and melanoma, while in patients with thyroid cancer, pancreatic cancer, and breast cancer, high *LEP* levels were associated with favorable outcomes. *LEPR* is a well-known receptor that triggers leptin-mediated biological effects. The correlation with cancer patient survival outcomes is also listed for comparison in Table 4. High *LEPR* expression is associated with poor outcomes in patients with thyroid cancer, breast cancer, cervical cancer, ovarian cancer, and gastric cancer. The clinical discrepancy observed among different research groups might result from the differences including the human races, case numbers enrolled in each cohort, endpoint setting, quality of care in hospitals, detection platforms (microarray vs next generation sequencing), random errors, and the involvement of complicated interaction networks contributing to the different outcomes in specific cancer types.

## 5. Summary and Perspectives

According to published findings and in silico analyses of clinical cancer databases, the expression of leptin and leptin receptors is found in many types of cancer. The signaling axis also plays a critical role in regulating several key processes in cancer progression, including cell proliferation, metastasis, angiogenesis, and drug resistance. We demonstrated the relative expression levels of leptin and leptin receptors in a pancancer panel. The differential RNA expression in specific cancer types suggests a potential alteration of upstream transcriptional activity and RNA stability that might be of value for further investigations in tumorigenesis and cancer progression. In the clinic, the discrepancy in observations among different research groups might result from the variations including the human races, case numbers enrolled in each cohorts, detection platforms and the involvement of complicated interaction networks contributing to the different outcomes in specific cancer types. We also listed controversial functional roles of leptin in liver cancer proliferation (Table 2), which might be due to the differences between humans and rats. In addition to the variation in the experimental procedures, it is possible that the leptin-mediated stimulatory or inhibitory effects are partly altered by other receptors which are still unknown in cancers, which might cause the discrepancies in leptin’s functional roles. Notably, the relative expression levels of leptin and leptin receptors were not uniformly distributed across the pancancer cohort. Head and neck cancer, peritoneal cancer, pancreatic cancer, cervical cancer, and breast cancer all expressed relatively high leptin but not leptin receptor, which suggests that the potential pathological involvement of other novel leptin receptors in tumors are required to be further explored and identified.

## Figures and Tables

**Table 1:** *LEP* and *LEPR* mutations in 5 major types of cancer.

	Lung			Liver	Colon		Glioblastoma	Ovarian
*LEP*	p14p	p23p	t37t	t48m				
	m89i	l101l	e102g	w121r	g8r	p23l	c7c	v134cfs*38
	h109l	v134a	g166v	l161q				
*LEPR*	a21s	l24f	p30t					
	44n	a51d	y64f					
	e65d	r92r	i123i					
	s146*	y155f	q182q					
	h185y	v229v	p264s					
	i281f	a284a	s303s		x14_splice	d124y		
	s320i	t330k	p338s		g179afs*35	v198a		
	t342a	g345w	g345v		s256y	s303s		
	s385t	n395qfs*4	e424*		x429_splice	l1094f		
	i432i	t451a	l464m		v430i	s541p		s60l
	c498y	r514s	d523y	t685s	w558*	r573h	a458v	t85i
	e565q	v600f	d602y	k994k	s595s	l598p	x799_splice	q463h
	y630f	m640v	k1135*	f1104y	v606v	r612c		s905*
	g651r	q677h	s686f		i814l	i845t		s1090i
	c687g	g696e	l715p		v846v	d921y		
	w758*	v792l	e805*		s927s	q1034h		
	q837k	l860i	w883r		k1074t	s1090r		
	k891k	a904t	l942v					
	s952c	q958*	e971d					
	l1020m	s1027r	l1061l					
	e1070*	g1081v	s1084*					
	k1086r	e1089*	r1114k					
	l1116l	g1131v						

Missense and frameshifts mutation (fs) were labelled in red color. Silent, nonsense (*) and splice site (_splice) mutations were labelled in blue color.

**Table 2:** Main effects of leptin/leptin receptor in different cancer types.

	Proliferation	Angiogenesis	Metastasis	Apoptosis	Drug Resistance
Ovarian Cancer	↑: [41]		↑: [38,39,40,42]		↑: (paclitaxel/docetaxel) [36]
Brain Tumor	↑: [45,46]	↑: [47,48]	↑: [45]		↑: (temozolomide) [49]
Breast Cancer	↑: [54,58]		↑: [53,55]		
Liver Cancer	↑: [60,63]		↑: [60,63]	↓: [63]	
	↓: [65] (rat)				
Colorectal Cancer	↑: [67,70]		↑: [67]		
			↓: [66]		
Lung Cancer	↑: [79,80]		↑: [76,77,80]		↑: (erlotinib) [78], (cisplatin) [79]
Pancreatic Cancer	↑: [82,87,88]		↑: [83]		↑: (gemcitabine) [86], (5-FU) [87]
Prostate Cancer	↑: [89]		↑: [89,90]	↓: [89]	
Gallbladder cancer	↑: [91]				
Myeloma					↑: (bortezomib) [92]
Chondrosarcoma		↑: [93,94]			

↑: increase.↓: decrease.

**Table 3:** Correlation of *LEP* with cancer patient survival.

Symbol	Cancer Type	Prognosis	Endpoint	*p*-Value	Case	Dataset	Method	Probe ID
*LEP*	Glioma	Poor	Overall survival	0.01	153	TCGA	RNA-seq	
*LEP*	Thyroid Cancer	Good	Overall survival	0.022	501	TCGA	RNA-seq	
*LEP*	Lung Cancer	Poor	Overall survival	0.0066	994	TCGA	RNA-seq	
*LEP*	Colorectal Cancer	Poor	Overall survival	<0.001	597	TCGA	RNA-seq	
*LEP*	Head and Neck Cancer	-	Overall survival	N.S.	499	TCGA	RNA-seq	
*LEP*	Stomach Cancer	-	Overall survival	N.S.	354	TCGA	RNA-seq	
*LEP*	Liver Cancer	N/A	Overall survival	N/A	365	TCGA	RNA-seq	
*LEP*	Pancreatic Cancer	Good	Overall survival	0.025	176	TCGA	RNA-seq	
*LEP*	Renal Cancer	Poor	Overall survival	0.001	877	TCGA	RNA-seq	
*LEP*	Urothelial Cancer	-	Overall survival	N.S.	406	TCGA	RNA-seq	
*LEP*	Prostate Cancer	-	Overall survival	N.S.	494	TCGA	RNA-seq	
*LEP*	Testis Cancer	-	Overall survival	N.S.	134	TCGA	RNA-seq	
*LEP*	Breast cancer	Good	Overall survival	0.0082	1075	TCGA	RNA-seq	
*LEP*	Cervical Cancer	-	Overall survival	N.S.	291	TCGA	RNA-seq	
*LEP*	Endometrial Cancer	-	Overall survival	N.S.	541	TCGA	RNA-seq	
*LEP*	Ovarian Cancer	Poor	Overall survival	0.014	373	TCGA	RNA-seq	
*LEP*	Melanoma	Poor	Overall survival	0.02	102	TCGA	RNA-seq	
*LEP*	Breast cancer	Good	Overall survival	0.037	1402	E-MTAB-365, E-TABM-43, GSE: 11,121, 12,093,	Array	207092_at
						12,276, 1456, 16,391, 16,446, 16,716, 17,705, 17,907,		
						18,728, 19,615, 20,194, 20,271, 2034, 20,685, 20,711,		
						21,653, 2603, 26,971, 2990, 31,448, 31,519, 32,646,		
						3494, 37,946, 41,998, 42,568, 45,255, 4611, 5327,		
						6532, 7390, 9195		
*LEP*	Ovarian cancer	-	Progression-free survival	N.S.	1435	GSE: 14,764, 15,622, 18,520, 19,829, 23,554, 26,193,	Array	207092_at
						26,712, 27,651, 30,161, 3149, 51,373, 63,885, 65,986,	RNA-seq	
						9891, TCGA (N = 565)		
*LEP*	Lung cancer	-	Post-progression survival	N.S.	344	CAARRAY, GSE: 14,814, 19,188, 29,013, 30,219,	Array	207092_at
						31,210, 3141, 31,908, 37,745, 43,580, 4573, 50,081,	RNA-seq	
						8894, TCGA (N = 133)		
*LEP*	Gastric cancer	-	Overall survival	N.S.	875	GSE: 14,210, 15,459, 22,377, 29,272, 51,105, 62,254	Array	207092_at

Survival data was collected from The Human Protein Atlas, Kaplan-Meier plotter databases. N.S.: no significance. N/A: not applicable.

**Table 4:** Correlation of *LEPR* with cancer patient survival.

Symbol	Cancer Type	Prognosis	Endpoint	*p*-Value	Case	Dataset	Method	Probe ID
*LEPR*	Glioma	-	Overall survival	N.S.	153	TCGA	RNA-seq	
*LEPR*	Thyroid Cancer	Poor	Overall survival	0.035	501	TCGA	RNA-seq	
*LEPR*	Lung Cancer	-	Overall survival	N.S.	994	TCGA	RNA-seq	
*LEPR*	Colorectal Cancer	-	Overall survival	N.S.	597	TCGA	RNA-seq	
*LEPR*	Head and Neck Cancer	-	Overall survival	N.S.	499	TCGA	RNA-seq	
*LEPR*	Stomach Cancer	-	Overall survival	N.S.	354	TCGA	RNA-seq	
*LEPR*	Liver Cancer	-	Overall survival	N.S.	365	TCGA	RNA-seq	
*LEPR*	Pancreatic Cancer	-	Overall survival	N.S.	176	TCGA	RNA-seq	
*LEPR*	Renal Cancer	-	Overall survival	N.S.	877	TCGA	RNA-seq	
*LEPR*	Urothelial Cancer	-	Overall survival	N.S.	406	TCGA	RNA-seq	
*LEPR*	Prostate Cancer	-	Overall survival	N.S.	494	TCGA	RNA-seq	
*LEPR*	Testis Cancer	-	Overall survival	N.S.	134	TCGA	RNA-seq	
*LEPR*	Breast cancer	Poor	Overall survival	0.016	1075	TCGA	RNA-seq	
*LEPR*	Cervical Cancer	Poor	Overall survival	0.0011	291	TCGA	RNA-seq	
*LEPR*	Endometrial Cancer	-	Overall survival	N.S.	541	TCGA	RNA-seq	
*LEPR*	Ovarian Cancer	Poor	Overall survival	0.0066	373	TCGA	RNA-seq	
*LEPR*	Melanoma	-	Overall survival	N.S.	102	TCGA	RNA-seq	
*LEPR*	Breast cancer	-	Overall survival	N.S.	1402	E-MTAB-365, E-TABM-43, GSE: 11,121, 12,093,	Array	207255_at
						12,276, 1456, 16,391, 16,446, 16,716, 17,705, 17,907,		
						18,728, 19,615, 20,194, 20,271, 2034, 20,685, 20,711,		
						21,653, 2603, 26,971, 2990, 31,448, 31,519, 32,646,		
						3494, 37,946, 41,998, 42,568, 45,255, 4611, 5327,		
						6532, 7390, 9195		
*LEPR*	Ovarian cancer	Poor	Progression-free survival	0.036	1435	GSE: 14,764, 15,622, 18,520, 19,829, 23,554, 26,193,	Array	207255_at
						26,712, 27,651, 30,161, 3149, 51,373, 63,885, 65,986,	RNA-seq	
						9891, TCGA (N = 565)		
*LEPR*	Lung cancer	-	Overall survival	N.S.	1925	CAARRAY, GSE: 14,814, 19,188, 29,013, 30,219,		207255_at
						31,210, 3141, 31,908, 37,745, 43,580, 4573, 50,081,	RNA-seq	
						8894, TCGA (N = 133)		
*LEPR*	Gastric cancer	Poor	Overall survival	<0.001	875	GSE: 14,210, 15,459, 22,377, 29,272, 51,105, 62,254	Array	207255_at

Survival data was collected from The Human Protein Atlas, Kaplan-Meier plotter databases. N.S.: no significance. N/A: not applicable.
